# Role of Socio-Demographic and Environmental Determinants on Performance of Community Health Workers in Western Kenya

**DOI:** 10.3390/ijerph182111707

**Published:** 2021-11-08

**Authors:** Fletcher Njororai, Daniel Ganu, Kogutu Caleb Nyaranga, Cholo Wilberforce

**Affiliations:** 1 Department of Health & Kinesiology, School of Community and Rural Health, The University of Texas at Tyler, 3900 University Blvd, Tyler, TX 75799, USA; 2 Department of Applied Sciences, School of Postgraduate Studies, Adventist University of Africa, Nairobi 00503, Kenya; ganud@aua.ac.ke; 3 Department of Public Health, School of Public Health and Biomedical Sciences, Masinde Muliro University of Science and Technology, KaKamega 50100, Kenya; kogutucal@gmail.com (K.C.N.); wilfavour77@vahoo.com (C.W.)

**Keywords:** performance, community health workers, health care, roles, Africa, challenges

## Abstract

***Background*:** The performance of community health workers remains an area of significant global focus. The role of community health workers in sub-Saharan Africa has evolved over time in response to changing health priorities, disease burdens, and workforce demands. Recently, Kenya revised its community health strategy in response to challenges faced with the implementation of grassroots primary health care initiative. Implementation of community health programs is often inconsistent, and they vary widely in many attributes. The purpose of this study was to explore factors influencing performance of community health workers in Vihiga County, Western Kenya in light of the political devolution. ***Methods*:** The study was a cross-sectional study design that involved a quantitative method of data collection. A sample of 309 participants was selected through cluster and simple random sampling. A self-administered and -structured questionnaire was used to gather data, s, and those who were not able to respond individually were guided by the research assistants. ***Results*:** The community health workers were 75.2% females and 24.8% males. Performance was significantly associated with not being employed, (OR = 2.4; 95% CI, 1.4–4.4), secondary education (OR = 0.7; 95% CI, 0.5–1.1), lack of conflict resolution mechanism (OR = 2.2; *p* = 0.017), lack of support (OR = 1.5; *p* = 0.03), and community health work not seen as important (OR = 1.5; *p* = 0.041). Poor communication skills were also more likely to influence performance of community health workers (OR = 0.5; *p* = 0.050) and poor road network (OR = 0.361; *p* = 0.000). ***Conclusions*:** These findings offer a deeper understanding of the interaction between CHWs contextual situations, structural challenges, and performance. Addressing influential factors of CHWs performance in multi-task settings is important in preventing overtaxing their work capacity and to maintain quality performance as countries move towards universal health coverage. Strategies for incentivizing, attracting, and sustaining men in CHWs is important to broaden perspectives about this critical role in society.

## 1. Introduction/Background

Globally, the critical role of community health workers in grassroots healthcare provision and health promotion is well documented, and their performance remains an area of significant global focus [1,2,3,4]. The role of CHWs has evolved over time in sub-Saharan Africa in response to the changing health care priorities, disease burdens, country policies, workforce capacity and shortages, and more [5,6,7]. While many definitions and names are given to describe this category of workers in different contexts, and their tasks vary widely, they have similarities across the board. Community health workers (CHWs) are defined as health workers carrying out functions related to healthcare delivery and health promotion at the grassroots level; they have some form of training in the context of intervention but have no formal professional or paraprofessional certificate or degree in tertiary education or health professions [1,2,4,8]. CHWs are a bridge between communities and formal health systems. Community health workers play a key role, especially in resource-limited settings, and bring on board some key attributes such as socio-cultural advantage as socio-cultural brokers who understand cultural norms, proximity, and ‘meeting people where they are’ among others [9]. Variations exist in structure, implementation, and types of CHWs programs in different parts of the world. There are variations in training, renumeration and practice settings, levels of knowledge, single or multiple health focus, administrative support, and health facility affiliation. An extensive report by the World Health Organization indicates that many factors may contribute to the performance of CHWs, including contextual factors, country health policies, intervention-related factors, competence and motivation, affiliation, and more [4]. In some countries, CHWs are salaried workers and an official part of the health sector, while in other countries, they are volunteers at the village level [1].

Kenya’s community health services were first introduced in 2006 by the government outlining the services in the country’s Community Health Strategy [6]. The primary goal is to expand access to health care for the community and whole population. The roles and responsibilities include activities that promote health, disease prevention, and control to reduce morbidity, mortality, and disability at all life cycle stages; provision of family planning services, maternal and child services, environmental hygiene and sanitation, home-based care, observed treatment, and some curative tasks depending on the context [6]. The foundation of their work includes promoting behavior change through health education, earlier case identification, and timely referral to trained health care providers. CHWs are community-selected community members who receive training of some kind for several weeks [10,11]. They carry out a wide range of activities at the household and community level and are often supervised by community health extension workers (CHEWs). CHEWs hold a certificate in public health and community nursing. The CHEWs carry out promotive, preventive, and curative tasks supported by fewer CHWs. Alongside the process of political devolution in Kenya, these changes shift responsibilities for health from the national to county level, subsequently influencing health priorities and health service implementation [6]. Under the new decentralized strategy, sub-counties are responsible for delivering health services and implementing health programs. Ref [6] posited that, cumulatively, these changes could potentially improve access to and utilization of health services significantly among vulnerable groups in Kenya; conversely, the reverse is true. There is a risk of the most disadvantaged groups having less access and use of health services.

The majority of CHWs in Kenya had been trained by non-governmental organizations (NGOs) in the context of primary health care from the early 80s. However, until only recently, there had been minimal government support and recognition for this work by the NGOs [12]. Recently, Kenya revised its community health strategy (CHS) in response to challenges faced with the implementation of grassroots primary health care initiative such as lack of funds to pay CHW salaries, high attrition, lack of accountability of voluntary CHWs, and high CHEWs workload [6]. Kenya’s interest and investment in close-to-community (CTC) health services has significantly grown, with substantial commitment from the government of Kenya, non-governmental organizations (NGOs), religious groups, and donors [6]. This has resulted in a wide variation of CHWs programs. The performance of these programs is often inconsistent. The outcomes vary. Sometimes referred to as *lay health workers*, CHWs mostly do work on a voluntary basis. According to [13] the attrition rate for male CHWs is higher due to the voluntary nature of the work and the societal norms of men being more responsible for family income. Additionally, a study by [14] reported that, in urban areas, CHWs face constrains in client follow-up due to migration and security issues. Policymakers need to address the role of CHWs in light of variations in context, program design, and quality, among others [4,6,15] The purpose of this study was to explore factors influencing performance of community health workers in Vihiga County, a rural county in Western Kenya considering the recent changes.

## 2. Methodology

### 2.1. Study Design

The study was a cross-sectional study design that involved quantitative method of data collection. This study focused on the factors influencing the performance of community health volunteers within the community units of Hamisi and Emuhaya sub-counties in Vihiga County, Kenya.

### 2.2. Study Area

Vihiga County is located in the Western region of Kenya, with its headquarters in Mbale. It lies in the Lake Victoria Basin between longitudes 34°30′ and 35°0′ E and between latitudes 0° and 0°15′ N. The county covers an area of 531.0 km^2^. The county’s altitude ranges between 1300 m and 1800 m above sea level. It slopes gently from East to West with undulating hills and valleys. The streams flow from northeast to southwest, draining into Lake Victoria. Vihiga County is located around 80 km northwest of Eldoret, around 60 km north of Kisumu, and approximately 350 km west of Nairobi City. The County has a total estimated population of 688,778 people.

The County has five administrative sub-counties, namely Hamisi, Emuhaya, Luanda, Sabatia, and Vihiga. The county is further subdivided into 11 divisions, 38 locations, and 131 sub-locations. Hamisi is the most expansive, with an area of 156.4 km^2^, Sabatia 110.9 km^2^, Vihiga 90.2 km^2^, Emuhaya 89.5 km^2^, and Luanda 84 km^2^.

### 2.3. Study Population

The study population were health workers in the cadre of community health volunteers (CHVs) and community health extension workers (CHEWs) as health care providers working in the community units within the health care system of Vihiga County. The county has one public county referral facility, Vihiga County Hospital at Mbale Township and Kaimosi Mission Hospital, a faith-based facility under the auspices of NCCK (National Council of Churches in Kenya). There were two sub-counties, level 4 facilities in Hamisi and Emuhaya, 18 health centres, 32 dispensaries, and 34 private and mission-based facilities. The average distance to the nearest facility is 5 km, and the county doctor to population ratio is 1:16,146, whereas the ratio of doctors to patients is 1:16,000. Further, the ratio of nurses to the population has been recorded as 103.4 per 100,000 populations. According to the World Health Organization, the recommended ratio is 250 healthcare workers, which includes physicians, nurses, and midwives, per every 100,000 population [16].

### 2.4. Sample Size

Slovin’s formula was used to arrive at the minimum sample size of 309 community health workers, some of whom were retired health care workers.

#### Sampling Method

The study adopted various sampling techniques at various stages accordingly, Vihiga County was picked from the 47 counties in Kenya using purposive sampling method because of its established community services in health care and the county has institutionalized the schemes of service for community health service. Cluster sampling was used to select on administrative locations where community health units occur. The community health units were selected using simple random sampling within the administrative location. All the community health workers in all the community units in the administrative locations were included as study subjects.

### 2.5. Data Collection Technique

A structured, self-administered questionnaire was used to collect quantitative data from the CHWs, and CHEWs. Three research assistants were recruited, trained, and taken through the questionnaire. They were all graduates with a bachelor’s degree in public health and were familiar with the topography of Vihiga County to help reach the sampled community units and health facilities. The primary objective of the training was to harmonize concepts on study design and content of the tools for the study before being used in collecting data. A structured questionnaire was self-administered to the respondents, and those who were not able to respond individually were guided by the research assistants. There was a lead field researcher who was responsible for the supervision of the completeness and consistency in data collection and field logistics.

### 2.6. Variables

#### 2.6.1. Dependent Variables

Performance was assessed by collecting data on availability, participation, and responsiveness of community health workers in community health work. Performance was then categorized as poor and better accordingly. 

#### 2.6.2. Independent Variables

Variables on factors influencing performance were demographic, and environmental factors, including lack of reference material, lack of conflict resolution mechanism, lack of support from the ministry, low literacy, little education, familiarity, community health workers seen as not important, lack of road network, poor transport, and community health work not readily accepted. 

### 2.7. Data Processing, Analysis, and Presentation

Data cleaning, entry, analysis, and interpretation of results were performed. Descriptive statistics were used to measure the frequency distribution of the role of CHWs, and univariate and generalized linear models were used to assess the extent to which various demographic characteristics influence the performance and engagement of community health volunteers. Binary logistic regression was used to explore the factors that affect performance and engagement of CHVs. Tables and bar charts were used for the presentation of the data. A *p*-value of less than 0.05 was considered statistically significant. The analysis was performed with the statistical package for social sciences, SPSS-software Version 23 (SPSS Inc., Chicago, IL, USA).

### 2.8. Ethical Considerations

Ethical clearance was obtained from the University of Eastern Africa, Baraton Ethics Review Committee (BERC) which is one of the local ethics review and approval bodies in the country. Thereafter, Vihiga County Administration, the department of medical services, and public health were informed of the study for approval. Community leaders were briefed on the purpose of the study, and their authority too was sought before the study commenced. Consent for the inclusion of subjects in this study was sought through a consent form that was read and presented to the respondents for their approval or disapproval before they accepted to participate in the study. Privacy and confidentiality of the information were assured; voluntary participation and withdrawal from the study at any stage without victimization were allowed, and the anonymity of the subjects was assured.

## 3. Results

### 3.1. Demographic Characteristics of Community Health Workers

Of all the community health workers, 75.2% were females and 24.8% males (see Table 1 below). Females were significantly overrepresented, with a female to male ratio of 3.0 (*x*^2^ = 4.0, *p* < 0.001). Although the sex ratio varied by age group, level of education, and marital status, a greater proportion of females are found in nearly every age group (Table 1). Sex differences among community health workers aged 18–20 years and 50 years and above were less prominent, with the respective ratios being 1.3 and 2.1. Community health workers who had completed secondary school had the highest sex ratio of 3.7; however, greater proportions of females were found among women who did not attain any formal education (100%). Those who had completed primary school were 1.5 times more likely to be female (OR = 1.5; 95% CI, 1.2 to 1.8, *p* < 0.001) than male in this category.

Although there was no significant difference in sex ratio among the clinical officers and trained community health workers 3.25 and 3.1, respectively (*x*^2^ =2.4, *p* < 0.29), trained CHWs were overrepresented (OR = 0.6; 95% CI, 0.5–0.7, *p* < 0.001) compared to clinical officers (OR = 1.0; 95% CI, 0.8–1.3, *p* = 0.9). Marital status significantly influenced the number of community health workers in diverse ways (*x*^2^ = 18.0, *p* < 0.001). There was an increased probability of community health workers being married, comprising 67.6% (OR = 0.7; 95% CI, 0.7–0.8, *p* < 0.001), compared to those who were separated. Married women and single ladies were also overrepresented, comprising 72.9% and 65.6%, respectively. Strikingly, there was an increased probability of community health workers who were divorced being men (OR = 0.5; 95% CI, 0.3–0.6, *p* < 0.001) rather than women (OR = 0.5; 95% CI, 0.9–0.1.1), with a proportion of 66.7% and 33.3%, respectively. The community health workers divorced (94.5%) and separated (100%) who participated in the study were predominantly female, with a sex ratio of 18.5.

### 3.2. Role of Community Health Workers

**Figure 1:**
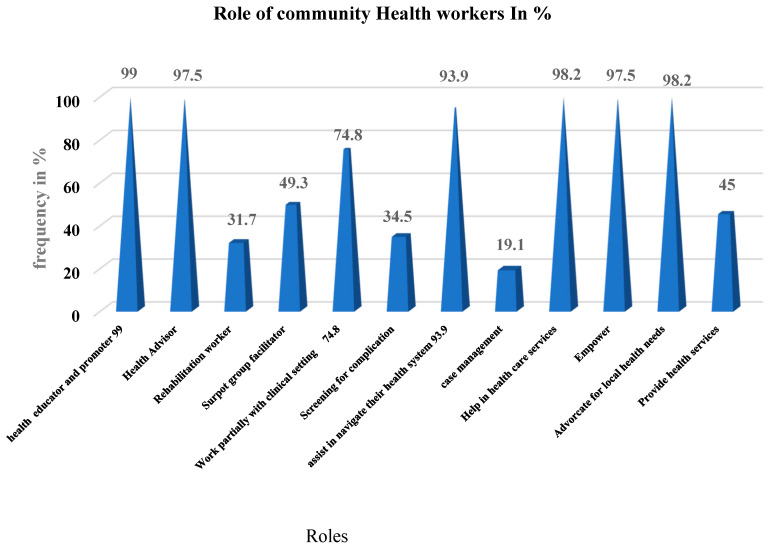
Role of community health workers.

The assessment of the role of community health workers showed that the roles included health educator and promoter, cited by 99% of the participants; these were followed by 98.2% of the respondents who said that community health workers help in the health care service. Community health workers’ role as health advisors and one that empowers community members was cited by 97.5% of the participants. Other roles were assisting the navigation of the health system (93.5%), working partially with the community (74.8%), and support group facilitator (49.1%); Case management accounted for the smallest proportion, representing 19.1 % (see Figure 1).

### 3.3. Demographics Characteristics and Performance of Community Health Workers

An attempt was made to assess whether those who participated on community health work differed on some characteristics from those who were not. Proportions of those who had better performance in community health work and poor performance were compared (Table 2). The age of community health workers influenced their participation and performance in community health programs significantly (*x*^2^ = 23.2, *p* < 0.001). The community health workers aged between 41–50 years were overrepresented in those with better performance (OR = 0.9; 95% CI, 0.8–1.2) than aged 51 and above and (OR = 0.7; 95% CI, (0.4–1.3)) with a proportion of 41%. There was increased participation and better performance of females (OR = 0.9; 95% CI, (0.8–1.1)) in community health work than of males, constituting over three quarters (75.9%) of the participants. There was increased probability of trained CHWs with better performance in community health work (OR = 0.9; 95% CI, 0.6–1.3) than clinical officers (OR = 1.0; 95% CI, 0.8–1.3). Married people, comprising 59.7% in community health work (OR = 0.7; 95% CI, 0.7–0.8), were more likely to perform better than those who were separated. Strikingly, there was increased performance of divorcees in community health work (OR = 1.1; 95% CI, 0.8–1.7); of the 39.2% of the study participants who were involved in the community health work, over half (58.7%) had completed secondary school, so that those who had completed secondary education had 70% probability of better performance in community health work (OR = 0.7; 95% CI, 0.5–1.1) than those who had no formal education. However, those who had completed primary education had 90% increased probability of performing better in community health work (OR = 0.9; 95% CI, 0.5–1.1) than those who had no formal education. Employment status influenced performance in community health work (*x*^2^ = 23.6, *p* < 0.001). Those who were not employed in anyway had 2.4 times increased likelihood of better performance in community health work (OR = 2.4; 95% CI, 1.4–4.4), while those who were self-employed were 1.6 times more likely to perform better in community health work (OR = 1.6; 95% CI, 1.1–1.6). Only 9.1% of those who were employed had better performance in community health work.

### 3.4. Environmental Factors Associated with Performance of Community Health Workers

Factors associated with performance of community health workers are highlighted in Table 3. Lack of conflict resolution mechanism, lack of support, and community health work not seen as important (OR = 1.5; 95% CI = 1.38–1.95; *p* = 0.041) had the highest odds. Poor communication skills were also more likely to influence performance of community health workers and poor road network.

## 4. Discussion

The productivity of the community health worker is mostly seen in the conditions under which they work. Provision of an empowering environment for CHWs is crucial for realizing utmost efficiency. Various interconnected inputs are needed for CHWs to become productive in their roles. This study described the performance of CHWs using two categorized indicators: socio-demographic and environmental variables. It was highlighted in the study that socio-demographic variables such as age, marital status, and educational and employment status are likely to influence performance of the CHWs in Vihiga County of Kenya. A similar finding was also noted by [17], where age, sex, level of education, and experience of the CHWs affected their performance positively. In the study, the age group between 41–50 showed better performance, which could be attributed to the experiences and skills gained over the years as a result of repetitive community health activities and continuous training. Females, however, showed slightly higher performance than their male counterparts.

Married CHWs showed higher performance than unmarried, but there was also an increased performance shown by the divorcees. It is also shown in the study of [18] that marital status is significantly associated with the job satisfaction of primary health care workers. The explanation of this could be attributed to family members or spouses who were available to lend a helping hand with household duties. Those who have completed secondary schools have higher performance than those with primary and no formal education. This result suggests that employing CHWs with higher education should be a significant criterion for better results. Our study demonstrated that CHWs who were employed showed 2.4 times increased likelihood of better performance than self-employed ones. The findings of our study indicated that socio-demographic determinants of the CHWs are important characteristics to consider in the CHW program.

The study also demonstrated that other factors leading to poor performance of the CHWs are lack of conflict resolution mechanism, lack of support from the government, lack of attached to the profession, poor communication skills, and poor road network. The results of the study highlight these five key contributions in understanding the performance of CHWs.

### 4.1. Lack of Conflict Resolution Mechanism

The CHWs performance was significantly associated with lack of conflict resolution mechanism (OR = 2.2; 95% CI = 1.9–2.4; *p* < 0.017). According to [19], conflict is one of the numerous challenges common to many health workers in the community. It was noted by [20] that unmanaged conflict is costly, not only in monetary and healthcare personnel cost but can also extend to affect the performance of health workers [21] found in his studies that health workers usually use provocative tactics in resolving issues in the workplace.

### 4.2. Lack of Support

In terms of lack of support, it was shown in the study of [22] that the physical condition at the health facilities of CHWs were challenging in South Africa. The CHWs were challenged in many complexes. Additionally, ref. [23] noted that monetary motivations and deprived working conditions were common among the challenges. Community health workers in Kenya require various support from the government, which include monetary support, capacity building on emerging health issues, a motorbike to facilitate their movement in the community, and descent facilities for their day-to-day activities.

### 4.3. CHWs Seen as Unimportant

The CHWs require recognition, support, and respect from the community and the formal health system for them to be effective in administering their duties. According to [22], a hierarchy played out in the health facilities, where the CHWs in South Africa were seen at the lowest level of the hierarchy. In South Africa, the CHWs are the lowest level in the health system, including their conditions of employment and their working environment, and they also lack the necessary equipment to perform their work safely. The kind of treatment they receive from the other healthcare workers indicated that their work was unrecognized and their contribution untrusted [22].

### 4.4. Poor Communication Skill

A study carried out by [24] found that the community health workers and their supervisors acknowledged that there was room for improvement in their communication capacity. Dealing with barriers perceived by the community requires communication skills in addition to updated knowledge. They realized that female CHWs found it difficult to discuss culturally sensitive topics such as family planning with the males in the community. The effects of poor communication in healthcare can have extremely serious consequences.

### 4.5. Poor Road Network

Community health workers (CHWs) often live in the community they serve. They spend much of their time traveling within the community, speaking to groups, visiting homes and health care facilities, distributing information, and otherwise connecting with local people. The study showed that a long journey coupled with a poor road network has a crippling effect on the performance of their work. Several studies have also shown that topographical challenges and the need to cover large distances hamper CHW performance [25] found that CHWs working with children health in Uganda realized that households with a proximity of 1 to 3 km from a health facility were 72% more likely to utilize CHW services compared to households residing within more than 3 km of a health facility. Therefore, proximity of CHWs and health facilities to their clients could affect utilization of CHW services. The study of [26] also showed that CHWs found it difficult to reach communities due to flooding, which hampered their performance. Therefore, health workers do not only require being motivated financially but also non-monetary motivation such as presence of a good road network can help to motivate CHW to perform well. In the rural setting of Vihiga County, the populations that CHWs serve often have limited access to transportation. Therefore, CHWs often travel to rural communities to provide services or conduct outreach.

An empowering work environment is a broad term to describe the inputs under which CHWs perform their duties. Full support for their work, respect from the community, good infrastructure, adequate communication skills, and proper communication skills are but few enabling factors to culture an empowering environment for high performance in their job. This study demonstrated that the CHW performance is affected by category of factors that emerge from the intricate background in which they work in Vihiga County. The findings offer a deeper understanding of the interaction between CHW’s personal and professional characteristic in the community.

## 5. Conclusions

CHW workers are key in guaranteeing the provision of primary health care services in many low-income settings. The overall results show that, in as much as these factors will demotivate the CHWs, their strong desire and feeling of getting the job done ensured their continued support and efforts even in the face of low motivation. This paper finds the following factors influencing the performance of CHWs in Vihiga performance in Kenya: lack of conflict resolution mechanism, lack of support, CHWs seen as unimportant, poor communication skills, and poor road network. These include newly growing factors that influence CHWs performance, which have not previously been identified in the in the country. These findings offer a deeper understanding of the interaction between CHWs personal and professional life and are also outside their control. Concentrating on the consideration of CHWs performance in multi-task settings is important in preventing overtaxing their work capacity and to maintain quality performance as countries move towards universal health coverage. Additionally, strategies for incentivizing, attracting, and sustaining men in CHWs is important to broaden perspectives about this critical role in society.

## Figures and Tables

**Table 1:** Demographic characteristics of community health workers.

	Sex					
Age	Male	Female	Total	OR (95%CI)	*p*-Value	Chi Square
18–20	4 (5.8)	5 (2.4)	9 (3.2)	1.7 (6.1–8.3)	0.001	4.79
21–30	4 (5.8)	21 (10.0)	25 (9.0)	1.3 (1.1–1.5)	0.013	
31–40	18 (26.0)	59 (28.2)	77 (27.7)	1.2 (1.0–1.4)	0.043	
41–50	28 (40.6)	85 (40.7)	113 (40.6)	1.1 (0.9–1.2)	0.349	
50 and above	17 (24.6)	37 (17.7)	54 (19.4)			
**Education level completed**						
None	0 (0)	8 (3.8)	8 (2.8)			
Primary	36 (52.2)	79 (37.8)	115 (41.4)	1.5 (1.2–1.8)	0.001	
Secondary	33 (47.8)	122 (58.4)	155 (55.8)	0.9 (0.8–1.3)	0.058	
Designation						
Clinical Officer	4 (5.8)	13 (6.2)	17 (6.1)	1.0 (0.8–1.3)	0.9	2.4
Trained CHV/CHEWs	65 (94.2)	196 (93.8)	261 (93.9)	0.6 (0.5–0.7)	<0.001	
**Marital status**						
Married	51 (27.1)	137 (72.9)	188 (67.6)	0.7 (0.7–0.8)	<0.001	
Single	10 (34.5)	19 (65.5)	29 (10.4)	0.7 (0.5–0.8)	<0.001	18.0
Divorced	6 (66.7)	3 (33.3)	9 (3.2)	0.5 (0.3–0.6)	<0.001	
Widowed	2 (5.1)	37 (94.9)	39 (14.0)	1.0 (0.9–1.1)	0.651	
Separated	0 (0.0)	39 (100)	39 (1.1)			

**Table 2:** Demographics characteristics and performance of community health workers.

	Performance in Community Health Work					
Characteristics	Better	Poor	Total	OR (95%Cl)	Chi Square	*p*-Value
Age					23.2	<0.001
18–20	1 (0.9)	8 (4.7)	9 (3.2)	0.7 (0.4–1.3)		
21–30	17 (15.6)	8 (4.7)	25 (9.0)	0.9 (0.7–1.1)		
31–40	37 (33.9)	40 (23.7)	77 (27.7)	0.9 (0.7–1.2)		
41–50	46 (42.2)	67 (40.2)	113 (41.0)	0.9 (0.8–1.2)		
51 and above	8 (7.3)	46 (27.2)	54 (19.4)	Ref		
	109 (39.2)	169 (61.8)	278 (100)			
**Sex**						
Male	34 (31.2)	33 (19.5)	67 (24.1)		4.9	0.027
Female	75 (68.8)	136 (80.4)	211 (75.9)	0.9 (0.8–1.1)		
	109 (39.2)	169 (69.2)	278 (100)			
Designation						
Clinical officer	9 (8.2)	6 (3.6)	15 (5.4)	0.9 (0.6–1.3)	2.1	0.351
Trained CHV	100 (9.2)	163 (96.4)	263 (94.4)	0.7 (0.5–1.1)		
	109 (39.20)	169 (69.8)	278			
**Marital status**						
Married	66 (60.6)	100 (59.1)	166 (59.7)	1.2 (1.0–1.3)	10.6	0.031
single	13 (11.9)	31 (18.3)	46 (16.5)	1.3 (1.1–1.7)		
Divorced	17 (15.6)	2 (1.2)	19 (6.8)	1.1 (0.8–1.7)		
Widowed	10 (9.2)	31 (18.3)	42 (15.1)	1.2 (0.9–1.6)		
Separated	3 (2.8)	2 (1.2)	5 (1.8)	ref		
	109 (39.2)	169 (69.8)	278 (100)			
**Level of education**						
None	1 (0.9)	3 (1.8)	4 (1.4)	Ref	0.5	0.97
Primary	44 (40.4)	69 (40.8)	113 (40.6)	0.9 (0.6–1.3)		
Secondary	64 (58.7)	97 (57.4)	161 (57.9)	0.7 (0.5–1.1)		
	109 (39.2)	169 (69.8)	278 (1000)			
**Employment status**						
Self employed	56 (51.4)	88 (52.1)	144 (51.8)	1.6 (1.1–2.3)	23.6	<0.001
Employed	10 (9.1)	48 (28.4)	58 (20.9)	1.6 (1.1–2.4)		
None	35 (32.1)	22 (13.0)	77 (27.7)	2.4 (1.4–4.4)		
Casual	8 (7.3)	11 (6.5)	19 (6.8)			
Total	109 (39.2)	169 (69.8)	278 (1000)			

**Table 3:** Factors associated performance of community health workers.

Factors	B	S.E.	Wald	df	Sig.	Exp (B)
Lack of road network	−1.018	0.247	17.016	1	0.000	0.361
Lack of reference material	0.025	0.412	0.004	1	0.951	1.025
Lack of conflict Resolution mechanism	0.800	0.336	5.684	1	0.017	2.225
Lack of support from the ministry	0.376	0.255	2.185	1	0.039	1.457
Low literacy	0.036	0.238	0.022	1	0.081	1.236
Little education	−0.099	0.174	0.321	1	0.501	0.906
Familiarity	−0.391	0.237	2.733	1	0.028	0.676
Seen as not important	−0.008	0.209	0.001	1	0.041	0.992
Not readily accepted	0.385	0.259	2.202	1	0.138	1.470
Misunderstanding	0.083	0.245	0.114	1	0.736	1.086
Poor transport	−0.014	0.316	0.0002	1	0.966	0.986
Poor communication skills	−0.576	0.297	3.750	1	0.050	0.562
Constant	1.866	1.147	2.648	1	0.104	6.463

## Data Availability

Data is contained within this article.

## References

[1] KoK M.C., Ormel H., Broerse J.E.W., Kane S., Namakhoma I., Otiso L., Sidat M., Kea A.Z., Taegtmeyer M., Theobald S. (2017). Optimizing the benefits of community health workers’ unique position between communities and the health sector: A comparative analysis of factors shaping relationships in four countries. Glob. Public Health.

[2] Lewin S., Munabi-Babigumira S., Glenton C., Daniels K., Bosch-Capblanch X., Van Wyk B.E., Odgaard-Jensen J., Johansen M., Aja G.N., Zwarenstein M. (2010). Lay health workers in primary and community health care for maternal and child health and management of infectious diseases. Cochrane Database Syst. Rev. (Online).

[3] Perry H.B., Hodgins S. (2021). Health for the People: Past, Current, and Future Contributions of National Community Health Worker Programs to Achieving Global Health Goals. Glob. Health Sci. Pract..

[4] Bhutta Z.A., Lassi Z.S., Pariyo G., Huicho L. (2010). Global Experience of Community Workers for Delivery of Health-Related Millennium Development Goals: A Systematic Review, Country Case Studies and Recommendations for Scaling Up.

[5] World Health Organization (WHO) (2010). Global Experience of Community Health Workers for Delivery of Health-Related Millennium Development Goals: A Systematic Review, Country Case Studies; and Recommendations for Integration into National Health Systems. Alliance Document. https://www.who.int/workforcealliance/knowledge/resources/chwreport/en/.

[6] Mccollum R., Otiso L., Mireku M., Theobald S., De Koning K., Hussein S., Taegtmeyer M. (2016). Exploring perceptions of community health policy in Kenya and identifying implications for policy change. Health Policy Plan..

[7] Perry H.B., Zulliger R., Rogers M.M. (2014). Community health workers in low-, middle-, and high-income countries: An overview of their history, recent evolution, and current effectiveness. Annu. Rev. Public Health.

[8] World Health Organization (WHO) (2007). Community Health Workers: What Do We Know about Them? A Policy Brief. https://www.who.int/hrh/documents/community_health_workers_brief.pdf.

[9] Maes K., Kalofonos I. (2013). Becoming and remaining community health workers: Perspectives from Ethiopia and Mozambique. Soc. Sci. Med..

[10] Ministry of Health (2006). Strategic Plan of Kenya Taking the Kenya Essential Package for Health to the Community A Strategy for the Delivery of Level One Services. https://www.communityledtotalsanitation.org/sites/communityledtotalsanitation.org/files/community_strategy.pdf.

[11] RoK (2006). Reversing the Trends. The Second National Health Sector Strategic Plan of Kenya. Taking the Kenya Essential Package for Health to the Community. A Strategy for the Delivery of Level One Services.

[12] Ndaraiya W.J., Wilberforce C. (2019). Role of health system practice on performance of Community Health Workers in Lurambi sub-County Kenya. Int. J. Res. Innov. Soc. Sci. (IJRISS).

[13] Olang’o C.O., Nyamongo I.K., Aagard-Hansen J. (2010). Staff attrition among community health workers in home-based care programmes for people living with HIV and AIDS in western Kenya. Health Policy.

[14] Mireku M., Kariuki M., McCollum R., Taegtmeyer M., Koning K.D., Otiso L. (2014). Report on the Context Analysis of Close-to-Community Health Service Providers in Kenya.

[15] Glenton C., Scheel I.B., Lewin S., Swingler G.H. (2011). Can lay health workers increase the uptake of childhood immunisation? Systematic review and typology. Trop. Med. Int. Health.

[16] World Health Organization Global Atlas of the Health Workforce. http://www.who.int/globalatlas/default.asp.

[17] Crispin N., Wamae A., Ndirangu M., Wamalwa G., Wangalwa G., Watako P., Mbiti E. (2012). Effects of Selected Socio-Demographic Characteristics of Community Health Workers on Performance of Home Visits during Pregnancy: A Cross-Sectional Study in Busia District, Kenya. Glob. J. Health Sci..

[18] Amoran O.E., Omokhodion F.O., Dairo M.D., Adebayo A.O. (2005). Job satisfaction among primary health care workers in three selected local government areas in southwest Nigeria. Niger. J. Med..

[19] Yu X., Davidhizar R. (2004). Conflict management styles of Asian and Asian American nurses: Implications for the nurse manager. Health Care Manag..

[20] Brinkert R. (2010). A literature review of conflict communication causes, costs, benefits, and interventions in nursing. J. Nurs. Manag..

[21] Osabuohein (2014). Industrial Conflict and Health Care Provision in Nigeria: An Interdisciplinary Discuss on the Human Condition. www.covenantuniversity.edu.ng.5/12/13.

[22] Watkins J.A., Griffiths F., Goudge J. (2021). Community health workers’ efforts to build health system trust in marginalised communities: A qualitative study from South Africa. BMJ Open.

[23] Maboko S., Hlongwana K., Mashamba-Thompson T.P. (2018). Factors influencing the motivation of community health workers in Vhembe district, Limpopo: Case study. S. Afr. Health Rev..

[24] Haq Z., Hafeez A. (2009). Knowledge and communication need assessment of community health workers in a developing country: A qualitative study. Hum. Resour. Health.

[25] Mukanga D., Tibenderana J.K., Peterson S., Pariyo G.W., Kiguli J., Waiswa P., Babirye R., Ojiambo G., Kasasa S., Pagnoni F. (2012). Access, acceptability and utilization of community health workers using diagnostics for case management of fever in Ugandan children: A cross-sectional study. Malar. J..

[26] Azad K., Barnett S., Banerjee B., Shaha S., Khan K., Rego A.R., Barua S., Flatman D., Pagel C., Prost A. (2010). Effect of scaling up women’s groups on birth outcomes in three rural districts in Bangladesh: A clusterrandomised controlled trial. Lancet.

